# Blood group AB is associated with reduced blood loss but also elevated cardiovascular mortality in aortocoronary bypass surgery

**DOI:** 10.1007/s11239-023-02934-3

**Published:** 2024-02-12

**Authors:** Franz Masseli, Arlinda Veseli, Marvin Pfohl, Jochen Hoch, Hendrik Treede, Wolfgang Schiller

**Affiliations:** 1 Department of Cardiac and Vascular Surgery, University Medical Center, Langenbeckstr. 1, 55131 Mainz, Germany; 2 Medical Faculty, University of Bonn, Sigmund-Freud-Straße 25, 53127 Bonn, Germany; 3 Department for Experimental Hematology and Transfusion Medicine, University Clinic Bonn, Sigmund-Freud-Straße 25, 53127 Bonn, Germany

**Keywords:** ABO blood-group system, Anticoagulants, Coronary artery bypass, Myocardial infarction, Von Willebrand factor

## Abstract

Patient blood group (BG) is predictive for von-Willebrand-factor (VWF) and Factor VIII variation. The clinical impact of this ABO-effect on blood loss, cardiovascular complications and outcome has been described for several patient cohorts. The aim of this study was to investigate the impact of patient BG on blood loss and outcome after coronary artery bypass surgery (CABG). Patient records, intraoperative data and perioperative transfusion records of 5713 patients receiving an on-pump CABG procedure between 05/2004 and 12/2018 were analyzed. A logistic regression model for death due to perioperative myocardial ischaemia (PMI) was developed from initially 24 variables by using an univariate and multivariate selection process. BG AB patients required less blood transfusions as compared to the other blood groups, especially in case of emergency operations. However, BG AB patients also had a higher mortality which was due to secondary cardiovascular complications. The impact of blood type on the rate of cardiovascular mortality was confirmed in the logistic regression model. BG AB patients have a worse outcome after CABG surgery due to an increased incidence of fatal cardiovascular complications. As perioperative myocardial ischemia due to graft occlusion appears to be the most likely explanation, stricter anticoagulation for BG AB patients should be discussed.

## Highlights

- Patient blood group (BG) is predictive for perioperative blood loss, thrombembolic complications and outcome.
- We investigated the impact of patient BG on blood loss and outcome after coronary artery bypass grafting (CABG).
- BG AB protects from perioperative hemorrhage but increases risk of cardiovascular complications after CABG.
- Stricter anticoagulation should be considered in BG AB patients.

## Introduction

Von-Willebrand Factor (VWF), a blood coagulation factor synthesized predominantly in endothelial cells and megakaryocytes is a major component of primary hemostasis by facilitating Gp1b/ GpIIb/IIIa mediated platelet adhesion as well as factor VIII recruitment and stabilization [1].

**Fig. 1:**
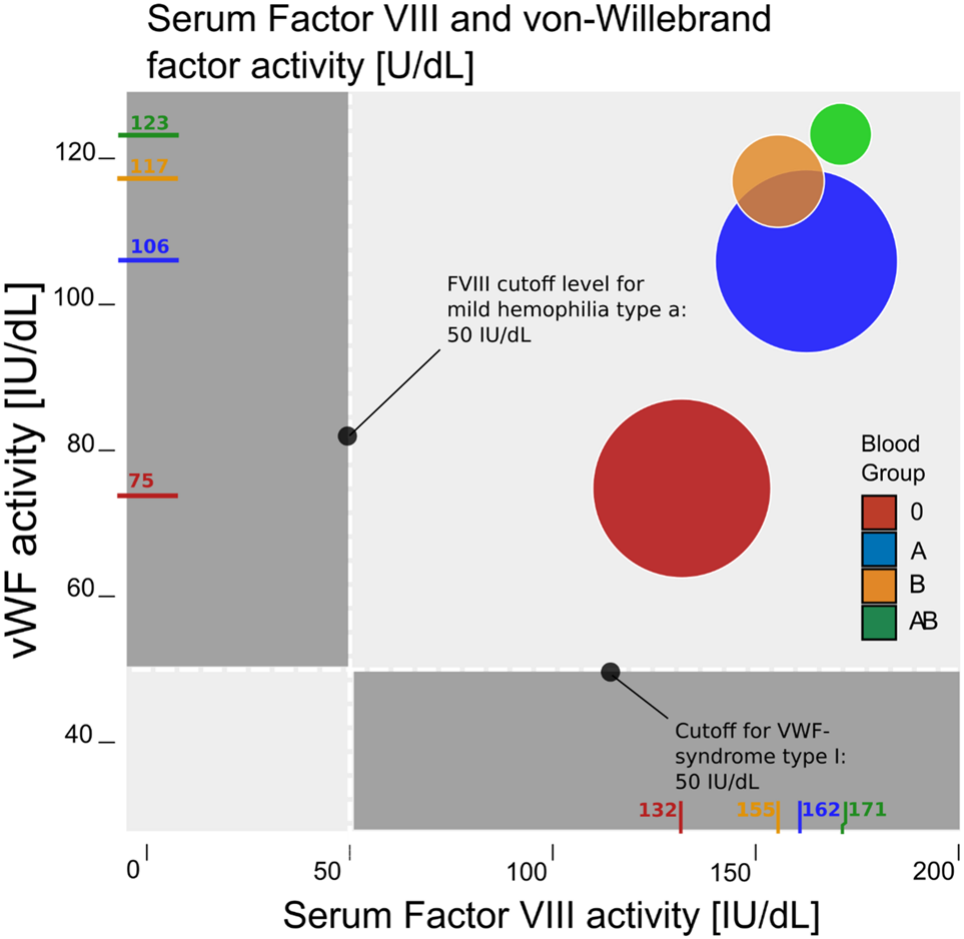
Variation of von-Willebrand factor (VWF) and Factor VIII levels between the ABO blood groups in normal individuals, modified from Gill et al. [3] and Vlot et al*.* [6]. Circles are sized according to the proportion of each blood group of the european population [8]. Dotted lines represent the diagnostic threshold levels for von-Willebrand ‘s disease type I and hemophilia type A

First described in 1924 by Erik von Willebrand, the qualitative or quantitative VWF deficiency known as the Von-Willebrand Syndrome is the most common of hereditary coagulation disorder [2]. When plasma levels of VWF were quantified in the 1970s, it was found that there was considerable variation between the human blood groups. Especially Blood Group (BG) O was found to have a 30% reduction in the circulating VWF levels as compared to Non-O. As the genotype of blood groups A or B is composed of heterogenous and homogenous alleles (AA, AO, BB, BO), these cohorts have intermediate levels of VWF. With no type O allele present, BG AB has the highest levels of plasma VWF [3–5]. As an excess of VWF is necessary in order to bind to and thus prevent degradation of factor VIII, serum concentrations of the latter are also lowest in BG type O individuals and higher within the Non-O groups [6]. As the blood group-specific range of VWF in BG O approaches the threshold for von-Willebrand-syndrome type I there is some difficulty separating pathology and normal variation in BG type O individuals [7] (Fig. 1).

Within the last decade, clinical implications of this type-O associated VWF deficiency have been illuminated: BG O as a determinate for lower VWF levels has been found to be protective against venous and arterial thrombotic and thrombembolic events such as myocardial infarction, deep vein thrombosis, cerebral ischemia and peripheral vascular disease [9–13]. On the other hand, BG O is also associated with an increased risk for significant bleeding in the context of stroke, oral anticoagulation, menorrhagia, gastrointestinal lesions and ECMO-therapy as well as a predictor for mortality after polytrauma [14–17].

Even though aorto-coronary bypass surgery (CABG) deals with a patient collective which is both at risk for ischaemic events such as perioperative myocardial ischaemia and stroke as well as for perioperative hemorrhage, the impact of this ABO-effect is mostly unexplored.

Aim of this study was to evaluate differences in blood transfusions and postoperative outcome related to the ABO blood groups in CABG patients based on a retrospective, single center analysis.

## Methods

### Patient selection and data collection

Approval by the internal review board of Bonn University was granted under the number 359/20 on September 2nd 2020. The requirement for patient consent was waived due to the retrospective nature of the study. Between 05/2004 and 12/2018, 5,722 consecutive patients who received isolated, on-pump coronary artery bypass surgery at our institution were included. Patients with a combination procedure such as CABG plus valve or vascular procedures, as well as off-pump procedures were excluded. ABO blood type as well as the number of perioperative transfusions of red blood cell concentrates (RBC), platelet concentrates (PC) and fresh frozen plasma units (FFP) were retrieved from our in-house blood bank database. Confirmed postoperative myocardial ischemia was defined as the clinical presentation of myocardial ischaemia which was confirmed by invasive imaging or characteristic Troponin elevation. Fatal malign arrhythmic events (ventricular fibrillation or pulseless electric activity in the absence of other causes such us tamponade or electrolyte disturbance) after CABG were defined as suspected postoperative myocardial ischaemia.

### Missing data

Missing data from the electronic records were collected, compared between groups and reported in Table 1. Items with missing entries above 10% were excluded from multivariable analysis. Missing entries included in the multivariable analysis were imputed by a multiple imputation algorythm using logistic regression from the MICE library for [18].

**Table 1:** Preoperative and intraoperative patient characteristics

Parameters	Blood groups				NA	p. value
	O (n = 2,180)	A (n = 2,591)	B (n = 667)	AB (n = 275)		
Preoperative						
Age [years]	67.8 sd: 9.42	68.35 sd: 9.35	67.46 sd: 9.43	68.5 sd: 9.53	–	0.06
Weight [kg]	83.46 sd: 16.36	83.21 sd: 15.9	83.72 sd: 16.03	81.89 sd: 15.43	-	0.41
Height [cm]	172.53 sd: 11.35	172.01 sd:10.25	171.51 sd: 12.72	170.84 sd: 13.71	–	0.03
Female [%]	20.41 (445)	21.57 (559)	20.69 (138)	24 (66)	–	0.49
Rhesus factor positive [%]	83.9 (1829)	84.18 (2181)	84.41 (563)	85.45 (235)	–	0.93
Left main vessel disease [%]	38.21 (833)	40.95 (1061)	40.78 (272)	35.27 (97)	0.42 (24)^a^	0.12
Pulmonal arterial hypertension [%]	2.16 (47)	1.66 (43)	2.7 (18)	2.18 (6)	3.96 (226)^a^	0.3
Aortic aneurysm [%]	3.03 (66)	3.09 (80)	2.55 (17)	2.91 (8)	3.17 (181)^a^	0.91
Arteriovascular disease: pAVK	15 (327)	16.02 (415)	15.89 (106)	15.64 (43)	3.33 (190)^a^	0.74
cAVK	14.31 (312)	15.13 (392)	12.89 (86)	17.09 (47)	2.43 (139)^a^	0.3
Neurologic deficit	2.52 (55)	2.28 (59)	2.25 (15)	1.46 (4)	–	0.78
Redo surgery	1.56 (34)	1.54 (40)	1.65 (11)	1.82 (5)	0.91 (52)a	0.95
Previous PCI	20.73 (452)	20.49 (531)	19.79 (132)	18.91 (52)	0.6 (34)a	0.9
ASA score on admission						
ASA < 3	22.48 (490)	23.08 (598)	25.34 (169)	22.18 (61)	–	0.48
ASA 3	65.09 (1419)	63.88 (1655)	60.87 (406)	64 (176)	–	0.26
ASA 4	9.4 (205)	9.92 (257)	10.64 (71)	11.27 (31)	–	0.63
ASA 5	0.73 (16)	0.66 (17)	1.05 (7)	0.73 (2)	–	0.71
Missing	2.29 (50)	2.47 (64)	2.1 (14)	1.82 (5)		0.92
Angina class on admission						
None	8.58 (187)	9.84 (255)	8.7 (58)	8 (22)	–	0.42
CCS2: upon strong exertion	9.36 (204)	10.04 (260)	7.95 (53)	6.91 (19)	–	0.19
CCS3: upon medium exertion	27.75 (605)	27.21 (705)	26.39 (176)	23.64 (65)	–	0.52
CCS4: Light exertion	22.61 (493)	22.58 (585)	24.89 (166)	27.64 (76)	–	0.17
CCS5: Resting Dyspnea	10.6 (231)	10.34 (268)	10.64 (71)	12 (33)	–	0.84
Missing	21.1 (460)	19.99 (518)	21.44 (143)	21.82 (60)		0.68
Dyspnea class on admission						
NYHA 1—no symptoms	5.28 (115)	5.87 (152)	5.25 (35)	4 (11)	–	0.58
NYHA 2—on strong exertion	20.46 (446)	20.57 (533)	20.09 (134)	19.27 (53)	–	0.97
NYHA 3—on light exertion	43.3 (944)	44.27 (1147)	42.73 (285)	44.73 (123)	–	0.84
NYHA 4—upon resting	9.86 (215)	9.34 (242)	10.49 (70)	10.18 (28)	–	0.78
Missing	21.1 (460)	19.95 (517)	21.44 (143)	21.82 (60)		0.67
LV ejection fraction						
50%+	51.15 (1115)	51.41 (1332)	52.47 (350)	52 (143)	–	0.94
30–50%	22.34 (487)	22.42 (581)	21.74 (145)	18.91 (52)	–	0.6
20–30%	3.94 (86)	4.79 (124)	3.15 (21)	4.36 (12)	–	0.23
< 20%	0.55 (12)	0.19 (5)	0.3 (2)	0.73 (2)	–	0.1
Mechanical assist device preop	0.78 (17)	0.81 (21)	1.05 (7)	0.73 (2)	–	0.89
High dose catecholamines	1.93 (42)	2.05 (53)	1.35 (9)	1.46 (4)	–	0.7
Emergency surgery	10.87 (237)	11 (285)	11.69 (78)	11.64 (32)	–	0.91
Intravenous inotropes	5.46 (119)	5.83 (151)	5.25 (35)	5.09 (14)	–	0.92
Intravenous nitroglycerol	13.95 (304)	13.86 (359)	17.24 (115)	16 (44)	–	0.12
Ventilated on admission	1.15 (25)	1.08 (28)	0.75 (5)	1.09 (3)	0.12 (7)^a^	0.89
Dialysis	1.47 (32)	1.16 (30)	0.9 (6)	2.18 (6)	20.65 (1180)^a^	0.3
Intraoperative						
Number of grafts	3.07 sd: 0.74	3.05 sd: 0.72	3.11 sd: 0.71	3.02 sd: 0.7	–	0.24
Cross clamping time (min)	69.31 sd: 22.26	68.94 sd: 21.78	70.83 sd: 21.43	67.01 sd: 20.31	–	0.08
Extracorporeal circulation time (min)	112.61 sd: 33.61	111.18 sd: 33.12	114.1 sd: 33.22	110.04 sd: 30.3	–	0.11
Minimum body temperature	33.86 sd: 2.8	33.93 sd: 2.24	33.83 sd: 2.46	33.62 sd: 3.65	–	0.25

Missing entries are reported either as separate entries or in the NA column

^a^Denotes a non-significant distribution of the missing entries with a p-value > 0.1

### Statistical analysis

Endpoints were perioperative administration of blood products and in-hospital mortality.

In univariate analysis, continuous variables were compared using a one-way ANOVA or Student’s t-test and reported as mean with standard deviation (SD) or standard error of the mean (SEM) as appropriate. Categorical variables were compared using the Fisher’s exact test. Selection for covariables in the multivariable logistic regression model was done by pre-selection with an univariate test for association with a threshold of p < 0.1 and an odds ratio (OR) > 2 [19]. Final selection was done by a stepwise reduction algorithm using the Akaike Information Criterion to compare models by successive iterations.

### Software

Statistical Analysis and graphical rendering was performed with the R statistical package version 3.6 (R Software foundation, Vienna, Austria, www.r-project.org) [20]. Logistic regression modeling was performed using the MASS library for R [21].

## Results

### Patient group

Between May 2004 and January 2019, 5722 consecutive patients underwent on-pump aortocoronary bypass surgery at our institution. Nine patients were excluded as their blood type could not precisely be determined. Of the remaining 5,713 patients, 2,180 (38.2%) belonged to BG O, 2,591 (45.40%) to BG A, 667 (11.70%) to BG B and 275 (4.80%) to BG AB.

Baseline characteristics as well as intraoperative parameters such as aortic cross clamping time, number of grafts and minimum body temperature were similar across the different blood groups (Table 1). A one-way ANOVA of the postoperative peak troponin levels revealed no significant differences between the groups suggesting an equal intraoperative ischemic burden (BG O: 12.97 ng/dL SEM: 0.98, A: 13.70 ng/dL SEM: 1.15, B: 11.98 ng/dL SEM: 1.57, AB: 15.96 ng/dL SEM: 3.52, F(3) = 0.483, p = 0.67).

### Perioperative blood product transfusions

**Fig. 2:**
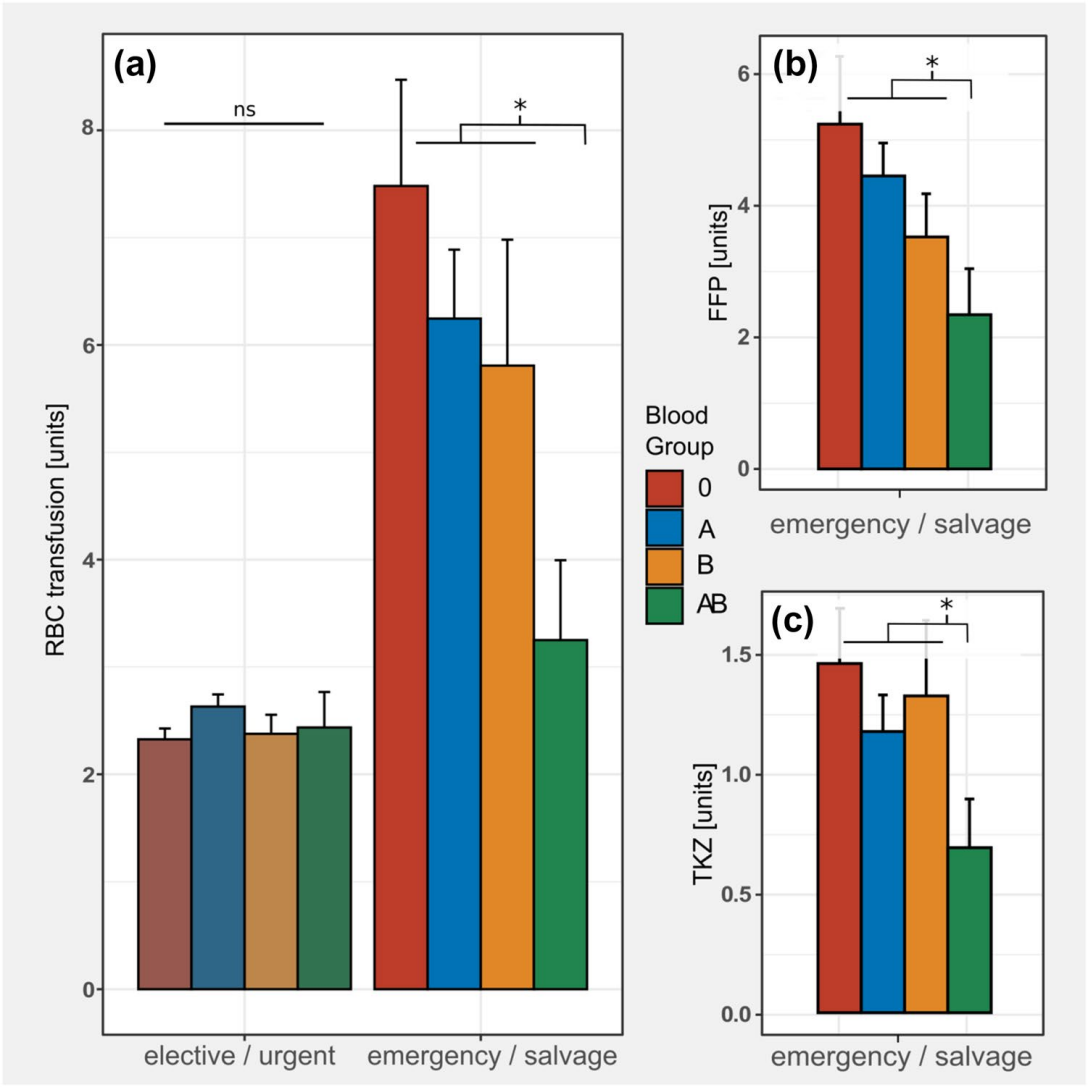
Perioperative blood product transfusions. **a** Transfusions of red blood cell concentrates (RBC) by blood group (BG) and urgency of the procedure. While the non-emergency group (elective/urgent) displayed no differences in the number of transfusions, significant differences between BG AB and Non-AB were observed in the emergency group. (emergency/salvage, 3.3 units, 0.74 SEM vs 6.3 units SEM: 0.6 and 7.5 units SEM: 1.0; p = 0.003 and p < 0.001). **b** and **c** In emergency patients, this trend was also observed with fresh frozen plasma units (FFP, 2.3 SEM: 0.7 vs. 4.5 SEM: 0.5 and 5.2 SEM: 1.1, p = 0.02) and platelet concentrates (PC: 0.7 SEM: 0.05 vs 1.3 SEM: 0.12, p = 0.033)

Overall, BG Non-AB patients required RBC transfusions more frequently as compared to BG AB (50.7% vs 44.4%, OR 1.29, p = 0.04). Regarding the amount of perioperative blood transfusions, there was no significant inter-BG difference in the patient cohort receiving non-emergency surgery (Fig. 2). However, in the patient subgroup receiving emergency surgery (n = 632), significant differences were observed between BG-AB vs. BG Non-AB. BG AB patients required less RBC units (3.3 SEM: 0.75 vs. 6.7 SEM: 0.52, p = 0.0003), FFPs (2.3 SEM: 0.75 vs 4.6 SEM: 0.49, p = 0.02) and PC (0.7 SEM: 0.20 vs 1.3 SEM: 0.12 p = 0.033) as compared to Non-AB patients. While BG type O patients also required more blood products overall as compared to Non-O, this differences did not reach significance (Fig. 2).

### In-hospital mortality

**Fig. 3:**
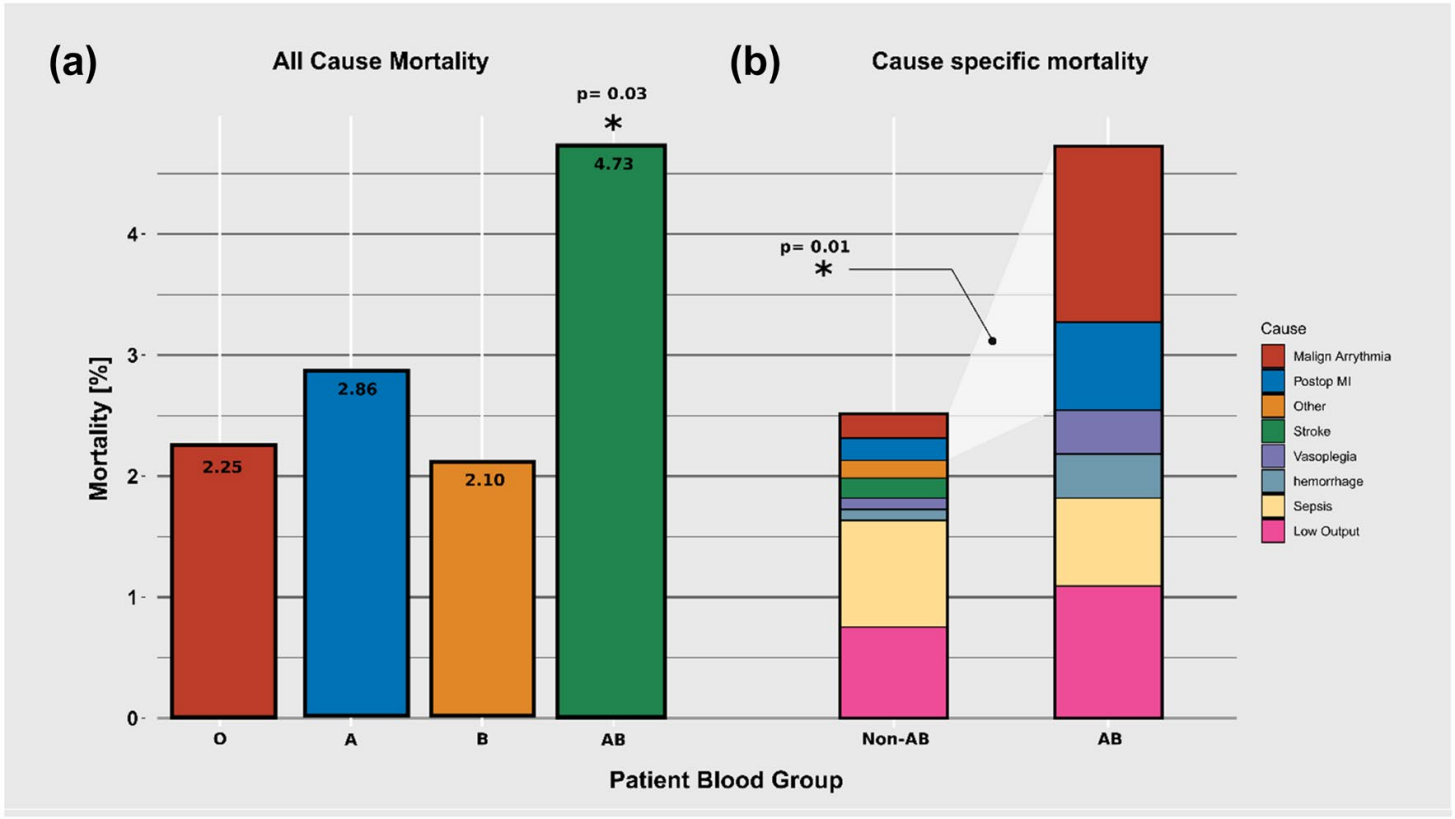
In-hospital mortality. **a** Blood group AB was associated with an increased mortality. **b** This excess mortality was due to increased proportion of malign arrhythmic events (ventricular fibrillation, pulseless electric activity) or confirmed postoperative myocardial infarctions (Postop MI)

Total in-hospital mortality was 2.6% (n = 150). While the non-AB groups had a similar outcome, BG AB patients had a significantly increased in-hospital mortality (4.73% vs 2.52%, OR 1.92 p = 0.032; Fig. 3a). Subgroup analysis of mortality by cause of death revealed that the increase in mortality was due to an elevated incidence in suspected as well as confirmed postoperative myocardial ischaemia (46.15% in BG-AB vs. 15.3% in Non-AB BGs, p = 0.013, Fig. 3b).

**Fig. 4:**
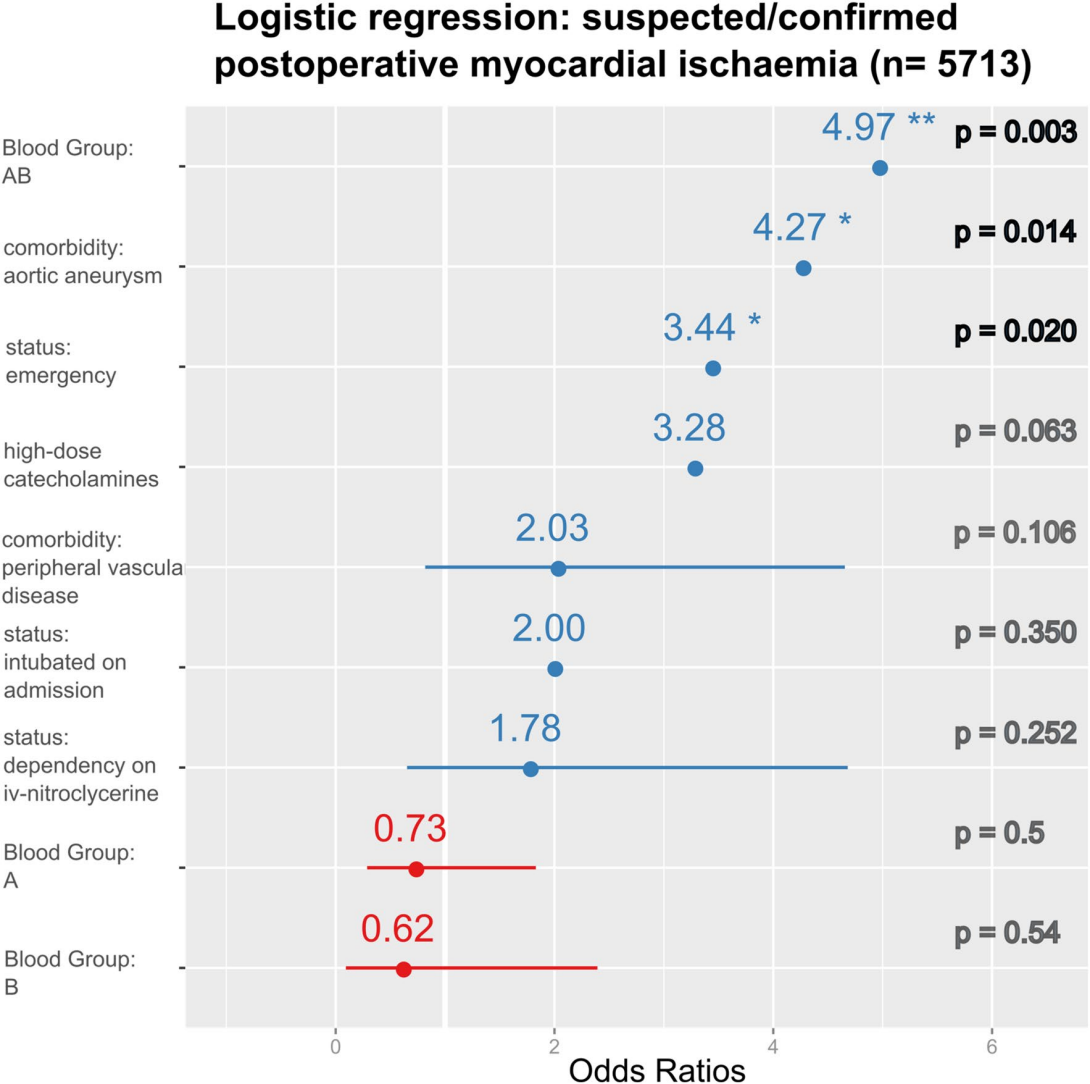
Results of the logistic regression analysis for the outcome suspected or confirmed postoperative myocardial ischaemia. Blood group AB, aortic aneurysm present and emergency status remain the only highly significant predictors, while high-dose catecholamine dependency had a borderline significance level

To further investigate the association of patient blood group and these fatal postoperative cardiovascular events, we developed a multivariable logistic regression model. From initially 24 possible covariables selected from the patient baseline characteristics (Table 1), we generated a model controlling for pre-operative status variables (patient intubated, preoperative intravenous nitroglycerine, high-dose catecholamines), vascular comorbidities (peripheral arterial occlusive disease, aortic aneurysm) and urgency of the operation. After controlling for these confounding factors, BG AB remained the strongest predictor for suspected/confirmed postoperative myocardial ischaemia (OR 4.97, p = 0.003, Fig. 4). Blood groups other than AB did not reach significance for predictive capacity.

## Discussion

In our single-center retrospective analysis of 5713 consecutive CABG patients, differences in blood product use and mortality were observed in relation to the ABO blood groups of the patients. BG AB was associated with reduced perioperative need for blood product transfusions, especially among patients receiving emergency/salvage CABG procedures. However, patients with BG AB had worse postoperative outcomes concerning in-hospital mortality compared to the Non-AB patients.

In several patient cohorts, BG O has been established as a risk factor for blood loss as compared to Non-O [14–17]. Although aortocoronary bypass surgery per itself is a procedure with an elevated risk for blood transfusions as compared to many other routine procedures, no inter-BG differences were found in non-emergency patients. In emergency CABG, which generally has a higher risk for blood loss as patients routinely enter the operation loaded with double platelet inhibitors and therapeutic doses of heparin in compliance with the current guidelines for acute myocardial infarction [22, 23], BG AB was associated with less transfusions in this study. Although a trend towards more frequent perioperative transfusions could be seen in BG O patients, the difference did not reach significance. Welsby et al. found in their study, that the blood loss between BG O and Non-O, investigated in a CABG collective of 877 patients, did not differ significantly [24]. This finding was confirmed in our observations only in those patients group that underwent elective or urgent CABG. However, in those patients undergoing CABG as emergency or salvage procedures, the patients with BG AB needed significantly less blood transfusions as compared to patients with BG Non-AB. This is well in concordance with a recent retrospective study in 17,058 patients undergoing cardiac surgical procedures in which Hansen et al. described a lower blood loss and transfusions of smaller mean volumes of all blood products in BG AB patients compared BG O patients [25].

While overall our postoperative outcome is comparable to the national average [26], the mortality for BG AB patients is almost twice as high as compared to the other blood groups. This increased mortality was due to an increase in either confirmed postoperative myocardial infarctions or malign arrhythmic events. We showed in a multivariate analysis that BG AB is the strongest predictor for death due to confirmed or suspected postoperative myocardial infarction with an odds ratio of 5 after controlling for relevant covariables. Therefore, BG AB patients have a worse postoperative outcome despite reduced blood loss as compared to the Non-AB group. As the increase in mortality is due to a proportional increase in suspected or confirmed postoperative myocardial ischemia, early postoperative myocardial infarction via graft occlusion might be a likely explanation for the surplus in mortality. Interestingly, in a retrospective study of 17,713 patients surviving CABG for at least one year Yavari et al. found hazard ratio of 1.19 (0.95% CI 1.00–1.42) for five year mortality in BG AB patients compared to BG O patients. However this effect did not persist after adjusting for most possible cardiovascular risk factors [27]. A recent comprehensive meta-analysis of 72 studies exploring the relationship between blood groups and ischemic strokes, myocardial infarction, and peripheral disease also revealed that patients with blood group AB had a 20% higher risk of myocardial infarction (p < 0.001) and 24% higher risk of ischemic stroke (p = 0.01) compared with other blood groups [28]. Our findings indicate that this increased risk for myocardial infarction in patients with blood group AB might be even more pronounced during procedures with activated coagulation system like during CABG. While the existent literature describes a protective role of BG O for acute coronary thrombosis via decreased VWF plasma levels, the reverse assumption has not been explored until now. As BG AB has the highest VWF levels of the ABO blood group system, an increased risk of thromboembolic events appears probable in light of our results. Regarding the ABO-effect, the scientific literature at large concentrates on the differences between BG O vs Non-O, as BG O is a common blood type. Since BG AB accounts for only approximately 5% of the European population, the previous studies lacked a sufficient sample size to examine the role of BG AB regarding postoperative thromboembolic events.

Current surgical guidelines recommend double platelet inhibition just for postoperative CABG patients with preoperative acute coronary syndrome and optional in patients with a higher ischemic risk due to coronary endarterectomy or off-pump surgery [29]. Accordingly the very recent guidelines of the European Society of Cardiology recommend that patients with acute coronary syndromes undergoing cardiac revascularisation should begin or resume double platelet inhibition therapy after surgery for at least 12 months [23].

The retrospective design of our single center study and reliance on data from the electronic patient records are clear limitations of our study. Furthermore, certain factors of interest such as the preoperative anticoagulation status could not be included in the study. Another limitation is the lack of data concerning VWF and factor VIII activity, precluding definitive conclusions on the mechanism how the BG AB influences the preoperative bleeding and mortality. Nevertheless, we believe that the observed results are robust because of the sample size and also because the observed effects confirm previously published data to a certain extent.

Our data strongly suggest that BG AB patients undergoing CABG procedures have some protection against excessive blood loss. Relative to patients with BG Non-AB, these patients are at an increased risk of adverse events related to thrombotic/thrombembolic complications. Although the current evidence on the relevance of the blood group does not yet support clinical decisions on anitcoagulation management in CABG patients in a systematic manner, the higher risk of thrombotic/thrombembolic complications in patients with BG AB should be taken into account. Since it is well established that BG AB patients have the highest VWF and factor VIII levels of all blood types, our conclusions are in line with the current status of research on the subject.

## References

[1] Eastlund T (1998). The histo-blood group ABO system and tissue transplantation. Transfusion.

[2] Nichols WC, Ginsburg D (1997). von Willebrand disease. Medicine (Baltimore).

[3] Gill JC, Endres-Brooks J, Bauer PJ, Marks WJ, Montgomery RR (1987). The effect of ABO blood group on the diagnosis of von Willebrand disease. Blood.

[4] Shima M, Fujimura Y, Nishiyama T, Tsujiuchi T, Narita N, Matsui T (1995). ABO blood group genotype and plasma von Willebrand factor in normal individuals. Vox Sang.

[5] Souto JC, Almasy L, Muñiz-Diaz E, Soria JM, Borrell M, Bayén L (2000). Functional effects of the ABO locus polymorphism on plasma levels of von Willebrand factor, factor VIII, and activated partial thromboplastin time. Arterioscler Thromb Vasc Biol.

[6] Vlot AJ, Koppelman SJ, Meijers JC, Dama C, van den Berg HM, Bouma BN (1996). Kinetics of factor VIII-von Willebrand factor association. Blood.

[7] O’Donnell J, Laffan MA (2001). The relationship between ABO histo-blood group, factor VIII and von Willebrand factor. Transfus Med.

[8] German Red Cross. Blood group distribution. https://www.blutspendedienst.com/blutspende/blut-blutgruppen/verteilung-der-blutgruppen. Accessed 08 Aug 2023.

[9] Dentali F, Sironi AP, Ageno W, Turato S, Bonfanti C, Frattini F (2012). Non-O blood type is the commonest genetic risk factor for VTE: results from a meta-analysis of the literature. Semin Thromb Hemost.

[10] Dentali F, Di Minno MND, Turato S, Crestani S, Ambrosino P, Bonfanti C (2014). Role of ABO blood group and of other risk factors on the presence of residual vein obstruction after deep-vein thrombosis. Thromb Res.

[11] Dentali F, Sironi AP, Ageno W, Crestani S, Franchini M (2014). ABO blood group and vascular disease: an update. Semin Thromb Hemost.

[12] Chen Z, Yang S-H, Xu H, Li J-J (2016). ABO blood group system and the coronary artery disease: an updated systematic review and meta-analysis. Sci Rep.

[13] Wu O, Bayoumi N, Vickers MA, Clark P (2008). ABO(H) blood groups and vascular disease: a systematic review and meta-analysis. J Thromb Haemost.

[14] Takayama W, Endo A, Koguchi H, Sugimoto M, Murata K, Otomo Y (2018). The impact of blood type O on mortality of severe trauma patients: a retrospective observational study. Crit Care.

[15] Mazzeffi M, Gupta R, Lonergan T, Pasrija C, Kon Z, Tanaka K (2017). ABO type and bleeding during adult ECMO. Intensive Care Med.

[16] Dentali F, Sironi AP, Ageno W, Bonfanti C, Crestani S, Frattini F (2013). Relationship between ABO blood group and hemorrhage: a systematic literature review and meta-analysis. Semin Thromb Hemost.

[17] Moeller A, Weippert-Kretschmer M, Prinz H, Kretschmer V (2001). Influence of ABO blood groups on primary hemostasis. Transfusion.

[18] van Buuren S, Groothuis-Oudshoorn K (2011). mice: Multivariate Imputation by chained equations in R. J Stat Softw.

[19] Bursac Z, Gauss CH, Williams DK, Hosmer DW (2008). Purposeful selection of variables in logistic regression. Source Code Biol Med.

[20] R Core Team (2013) R: a language and environment for statistical computing. R Foundation for Statistical Computing, Vienna. http://www.R-project.org. Accessed 08 Aug 2021.

[21] Venables WN, Ripley BD (2002). Modern applied statistics with MASS.

[22] Prejean SP, Din M, Reyes E, Hage FG (2018). Guidelines in review: comparison of the 2014 AHA/ACC guideline for the management of patients with non-ST-elevation acute coronary syndromes and the 2015 ESC guidelines for the management of acute coronary syndromes in patients presenting without persistent ST-segment elevation. J Nucl Cardiol.

[23] Byrne RA, Rossello X, Coughlan JJ, Barbato E, Berry C, Chieffo A (2023). 2023 ESC Guidelines for the management of acute coronary syndromes. Eur Heart J Acute Cardiovasc Care.

[24] Welsby IJ, Jones R, Pylman J, Mark JB, Brudney CS, Phillips-Bute B (2007). ABO blood group and bleeding after coronary artery bypass graft surgery. Blood Coagul Fibrinolysis.

[25] Hansen SM, Sprogøe U, Möller S, Andersen C (2021). ABO blood group is related to bleeding in cardiac surgery. Acta Anaesthesiol Scand.

[26] Meinertz T, Hamm C, Schlensak C, Fleck E, Cremer J, Stiller B, et al (2018) Deutscher Herzbericht 2017 29. Bericht/Sektorenübergreifende Versorgungsanalyse zur Kardiologie, Herzchirurgie und Kinderherzmedizin in Deutschland. Deutsche Herzstiftung, Frankfurt am Main

[27] Yavari N, Masoudkabir F, Landy MG, Pashang M, Sadeghian S, Jalali A, Shafiee A (2022). Effect of Different blood groups on long-term outcomes of surgical revascularisation. Heart Lung Circ.

[28] Lilova Z, Hassan F, Riaz M, Ironside J, Ken-Dror G, Han T, Sharma P (2023). Blood group and ischemic stroke, myocardial infarction, and peripheral vascular disease: A meta-analysis of over 145,000 cases and 2,000,000 controls. J Stroke Cerebrovasc Dis.

[29] Sousa-Uva M, Head SJ, Milojevic M, Collet JP, Landoni G, Castella M (2018). 2017 EACTS Guidelines on perioperative medication in adult cardiac surgery. Eur J Cardiothorac Surg.

